# Lipoprotein metabolism and inflammation in healthy young subjects – exploring the postprandial and postabsorptive phases following intake of a standardized meal

**DOI:** 10.1016/j.athplu.2026.100572

**Published:** 2026-06-03

**Authors:** Silje-Marie Jensen, Kirsten B. Holven, Stine M. Ulven, Åslaug Matre Anfinsen, Jutta Dierkes, Vegard Lysne, Jacob J. Christensen

**Affiliations:** a Department of Nutrition, Institute of Basic Medical Sciences, University of Oslo, Oslo, Norway; b Norwegian National Advisory Unit on Familial Hypercholesterolemia, Oslo University Hospital, Oslo, Norway; c Centre for Nutrition, Department of Clinical Science, University of Bergen, Bergen, Norway; d Department of Heart Disease, Haukeland University Hospital, Bergen, Norway

**Keywords:** Non-fasting blood sampling, Lipid profile, Postprandial metabolism, Lipoprotein, Inflammatory response, NMR metabolomics, ELISA, Age and sex differences, Standard breakfast meal, Healthy subjects

## Abstract

**Background and aims:**

Commonly measured cardiometabolic biomarkers have not been systematically characterized across the long-term continuum of the postprandial and postabsorptive phases. The aim of the present study was to evaluate how the exact time since last meal impacts test results for lipoprotein particles and inflammatory biomarkers.

**Methods:**

We used data from a trial where 34 normal-weighted males and females aged 20-30 y were included. Subjects fasted 12 h, had blood sampled at baseline, then consumed a standardized, habitual breakfast meal, and had blood sampled 13 more times over the next 24 h. NMR metabolomics was used to quantify lipoprotein subclasses and various biomarkers, and ELISA to analyze VCAM-1, ICAM-1, E-selectin, and IL-6. We characterized the postprandial and postabsorptive responses using visualizations and non-linear mixed effects models.

**Results:**

Six VLDL subclasses increased by 11-429% within 2-4 h postprandially before returning to baseline levels, followed by another increase 8-10 h after intake of the breakfast meal. IDL and three LDL subclasses increased around 10-12% over 24 h. Four HDL subclasses showed an inverse association with VLDL subclasses, reaching their lowest levels about 1 h after the meal and peaking after 8-10 h with a 5-15% increase from baseline. Participants had an average increase of 325% in IL-6 10 h after breakfast, while other inflammatory biomarkers showed little change over time. For most biomarkers, males generally exhibited higher baseline concentrations compared to females, however the responses over time remained similar.

**Conclusions:**

Baseline levels and time of return to baseline varied between individuals, with some sex-specific differences in responses. TG-rich lipoproteins and VLDL increased postprandially and normalized by 12-24 h, whereas IDL and LDL showed minor early changes but increased by 24 h. IL-6 increased markedly during the first 10 h and returned to baseline by 12-24 h, while other inflammatory markers changed little.

## Highlights

• Time since last meal alters lipoprotein and inflammatory test results.
• Six VLDL subclasses rose 11-429% within 2-4 h after breakfast.
• VLDL normalized, then a second peak occurred 8-10 h postprandially.
• IL-6 increased ∼325% around 10 h; other inflammatory markers changed little.
• Sex differences in baseline, but temporal responses were generally similar.

## Graphical abstract

Time-resolved variation in lipoprotein subclasses and inflammatory biomarkers following intake of a standardized breakfast in healthy young adults. Abbreviations: CVD, cardiovascular disease; ELISA, enzyme-linked immunosorbent assay; h, hours; HDL, high-density lipoprotein; ICAM-1, intercellular adhesion molecule-1; IL-6, interleukin-6; IDL, intermediate-density lipoprotein; LDL, low-density lipoprotein; NMR, nuclear magnetic resonance; VCAM-1, vascular cell adhesion molecule-1; VLDL, very low-density lipoprotein; y, years.

**Figure:**
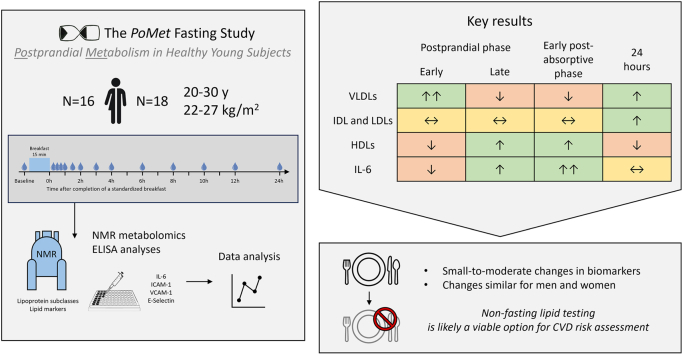

## 1 Introduction

Blood biomarker concentrations vary dynamically over the day, and for nutrition-related biomarkers, a principal driver of this variation is recent food intake – both meal composition and time since ingestion markedly influence measured concentrations [1]. Knowledge about the diurnal and postprandial variability for biomarkers therefore has direct implications for sampling strategy and the interpretation of clinical and epidemiological measurements [1,2].

Cardiometabolic biomarkers, including circulating lipids and inflammatory biomarkers, are responsive to feeding and fasting. For example, because triglycerides increase in the postprandial phase, lipids have traditionally been measured in fasting blood samples commonly defined as ≥ 6-8 h since last meal) [1]; however, non-fasting blood sampling is now increasingly recommended for lipid testing [[3], [4], [5], [6]]. Most studies that have characterized postprandial biomarker changes have sampled within the early postprandial window (roughly the first 6-8 h) [[7], [8], [9], [10], [11], [12], [13]], while fewer have tracked later, postabsorptive dynamics beyond this period [14,15]. Data from extended sampling is important to determine when concentrations return to postabsorptive baseline levels, and to quantify how much postprandial and postabsorptive effects may bias clinical or research measurements. Based on a controlled study with repeated sampling over 24 h, we recently investigated postprandial and postabsorptive changes using clinical chemistry, for lipids, ketones and acylcarnitines [14], and for amino acids, one-carbon metabolites and B-vitamin biomarkers [15]. We found for example that the concentrations of HDL-C and LDL-C decreased to approx. 2 h (−4%) while TG peaked at 3 h (+27%); importantly also, concentrations at 8-12 h often differed from 24 h, illustrating that “fasted” is a continuous – not binary – state.

Moreover, most prior work has relied on a relatively small panel of conventional assays. In contrast, high-dimensional platforms such as NMR metabolomics enable detailed quantification of lipoprotein particle subclasses and other biomarkers [16], but systematic data on how NMR-derived lipoprotein profiles and related inflammatory markers change across the full, 24-h, postprandial-to-postabsorptive continuum are sparse.

To address these gaps, using state-of-the-art NMR metabolomics together with targeted ELISA analyses, we aimed to describe the sex-specific dynamic variation in the concentrations of lipoproteins and inflammatory markers during the postprandial and postabsorptive phases. We used data from a trial on 34 young and healthy subjects who had blood drawn after a 12-h overnight fast, and then at 13 subsequent time points during the next 24 h following ingestion of a standardized, habitual breakfast meal.

## 2 Subjects and methods

### 2.1 Study design, setting and participants

In the present study, we investigated postprandial and postabsorptive changes in lipoprotein subclasses and inflammatory markers using data from a recently conducted trial. The study design has been described in detail previously [15].

Eligible participants were healthy men and women aged 20-30 years (birth years 1991-2001), with BMI 22-27 kg/m^2^, and without significant weight change (>5%) within three months before the study visit. Participants were recruited through social media and posters in the local area in Bergen, Norway.

In brief, participants consumed a standardized evening meal at 20:00 p.m. and then fasted overnight (approx. 12 h). The following day, participants met at the Research Unit for Health Surveys at the University of Bergen, Norway, and had clinical measurements and blood samples taken at baseline. Participants were then instructed to consume a standardized breakfast in precisely 15 min. They were thereafter subjected to frequent blood sampling for 24 h with a total of 13 additional blood samples drawn from each participant (12 blood samples first 12 h while being supervised in the research unit, and one blood sample after non-supervised 12 h at the following morning, after 24 h). Fasting during the unsupervised hours was confirmed by increased ketone bodies measured throughout the study and after 24 h [15].

During the 24 h of blood sampling, the participants were fasting but free to drink water. The standardized meal was composed to mimic a habitual Norwegian breakfast and consisted of two slices of whole grain bread with butter and low-fat cheese, one slice of whole grain bread with strawberry jam, and one glass of orange juice. The composition of the meal was 520 kcal, of which 20,5 g (36 E%) was fat, 59,4 g (46 E%) was carbohydrates, 20,5 g (16 E%) was protein, and containing 5 g (1,9 E%) fiber.

### 2.2 Assessment of lipoproteins with NMR metabolomics

Concentrations of lipoproteins and other biomarkers were quantified by using high-throughput proton NMR spectroscopy metabolomics (2020 Algorithm; Nightingale Health, Helsinki, Finland). The platform provides 250 biomarkers per sample, including lipoproteins, lipids, fatty acids, and small molecules such as amino acids, ketones, and glycolysis metabolites [16]. In the present work, we included 14 lipoprotein subclasses, the inflammatory biomarker Glycoprotein acetyls (GlycA), and lipids, cholesterol measurements, apolipoproteins, phospholipids, and lipid concentration within the subclasses. The lipoprotein subclasses were classified based on average particle sizes (in nanometers). NMR-measured levels of ketones were included as a measure of compliance.

For completeness of the standard lipid panel, we included in our analyses both “Clinical LDL-C” and “LDL-C”. The LDL-C measure labeled Clinical LDL-C is calibrated against direct LDL-C assays used in routine clinical chemistry, and it broadly corresponds to LDL-C levels obtained using the Friedewald equation [17]. In contrast, the variable simply labeled LDL-C in the Nightingale panel represents “size-specific LDL-C″, that is, the sum of cholesterol content across LDL subclasses defined by their size. Note that the size-specific LDL-C is always lower than clinical LDL-C because the underlying operational definitions of LDL differ [18].

### 2.3 Assessment of inflammatory response with ELISA

We used ELISA to analyze the inflammatory markers Vascular Cell Adhesion Molecule 1 (VCAM-1), Intercellular Adhesion Molecule 1 (ICAM-1), Endothelial Adhesion Molecule 1 (E-selection), and Interleukin 6 (IL-6). ELISA analyses were performed at the Department of Nutrition, UiO, using commercially available ELISA Duo and Quantikine kits (R&D Systems, Minneapolis, USA). The following kits were used: Human VCAM-1/CD106 DuoSet ELISA (DY809), Human ICAM-1/CD54 DuoSet ELISA (DY720), E-Selectin/CD62E DuoSet ELISA (DY724), Human IL-6 Quantikine HS ELISA (HS600C). Intra-assay CV was between 4.4 and 5.0 % for all assays.

### 2.4 Statistical analyses

Data analyses were performed in R version 4.2.3 [19] using the RStudio IDE (https://posit.co/) and the tidyverse framework [20].

Data are presented with counts (and percentages) for categorical variables, or geometric mean (gMean) and geometric standard deviations (gSD) or geometric confidence intervals (gCI) for continuous variables. Results were presented as visualizations of metabolite concentrations as a function of time, with gMean and gCI superimposed on top of individual-level data. gCI was calculated by using the following formulas:

1) $gSE=gSD\frac{1}{\sqrt{n}}$
2) $95\;\%\;CI\;lower\;limit=\frac{gMean}{{gSE}^{1.96}}$
3) $95\;\%\;CI\;upper\;limit=gMean\times {gSE}^{1.96}$

We also described the lowest and highest (peak) points on the curve trajectories, relative to the baseline level.

For each biomarker, we fitted non-linear mixed effect models using the lme4lmer function with time, sex, BMI, and age as covariates (to account for potential group differences), plus a random intercept for ID to account for dependency between each individual's 14 data points. Time was modeled using a B-spline function using the splines::bs function, and the interaction terms for sex and time, age and time, and BMI and time were also included in the model. We extracted β coefficients, 95 % CIs and P values for the sex differences (males vs. females), and used analysis of variance (ANOVA) to derive global P values for the non-linear terms for the sex by time interaction (that is, the sex differences in response over time). Standard deviation (SD)-normalized coefficients and P values for sex are shown in the figures (normalized to enable comparison between panels), while full models on original scale are shown in Table S3, including coefficients and P values for BMI and age.

The sample size was calculated using an accuracy-in-parameter-estimation approach to estimate the majority of measurements within a multiplicative margin-of-error of 10 %. The calculations are described in the main article [15].

### 2.5 Ethical approvals

The present study was registered at ClinicalTrials.gov (NCT number 04989478), conducted in accordance with the Declaration of Helsinki, and approved by the Regional Committee for Research Ethics for the Western part of Norway, located at the University of Bergen (REK 236654). Participants signed a consent form prior to engaging in the study and had the chance to withdraw from the study at any time.

## 3 Results

### 3.1 Baseline characteristics

We included data from all 34 participants, of which 18 (52 %) were males and 16 (48%) females. 33 participants completed all measurements, and one participant (female) completed the first 2 h (7 measurements). In total we received 469 blood samples. Compared to females, males were older (26.2 vs. 24.6 years) and had slightly higher BMI (23.9 vs. 23.1 kg/m^2^) (Table 1). All subjects were normolipidemic at baseline; while males had slightly higher levels of LDL cholesterol, females had higher levels of HDL cholesterol.

**Table 1:** Baseline characteristics of the study population.

	Overall, N = 34	Females, N = 16^a^	Males, N = 18
***Clinical***			
Age, yrs	25.3 (1.1)	24.5 (1.1)	26.1 (1.1)
< 25.8 yrs	17 (50%)	9 (56%)	8 (44%)
≥ 25.8 yrs	17 (50%)	7 (44%)	10 (56%)
Weight, kg	73.4 (1.2)	65.2 (1.1)	80.9 (1.1)
Height, cm	176 (1.1)	168 (1.1)	184 (1.0)
BMI, kg/m^2^	23.5 (1.1)	23.1 (1.1)	23.8 (1.1)
< 25, kg/m^2^	26 (79%)	13 (87%)	13 (72%)
≥ 25, kg/m^2^	7 (21%)	2 (13%)	5 (28%)
***Biochemical***			
Glucose, mmol/l	5.3 (1.1)	5.1 (1.1)	5.4 (1.1)
Insulin, mIU/L	4.5 (1.9)	4.4 (2.2)	4.5 (1.8)
HbA1c, mmol/mol	31 (1.1)	31 (1.1)	32 (1.1)
LDL-C, mmol/L	2.5 (1.3)	2.4 (1.2)	2.6 (1.4)
HDL-C, mmol/L	1.6 (1.2)	1.7 (1.3)	1.5 (1.2)
Triglycerides, mmol/L	0.9 (1.5)	0.8 (1.5)	0.9 (1.5)
CRP, mg/L	0.9 (2.6)	1.4 (2.6)	0.6 (2.1)

Data are presented as geometric mean (geometric SD) for continuous variables and as count (percentage) for categorical variables. gSD indicates how much the data varies multiplicative from the gMean; for example, for overall age, most data are covered by the interval [25.3/1.13 – 25.3∗1.13] = [22.4 – 28.6] yrs. Note that potential differences in age and BMI between sexes are accounted for in the modeling.

Abbreviations: BMI; body mass index, CRP; C-reactive protein, HbA1c; glycated hemoglobin, HDL-C; cholesterol in high-density lipoprotein, LDL-C; cholesterol in low-density lipoprotein.

a n = 15 for weight and BMI.

### 3.2 Standard lipid panel

**Fig. 1:**
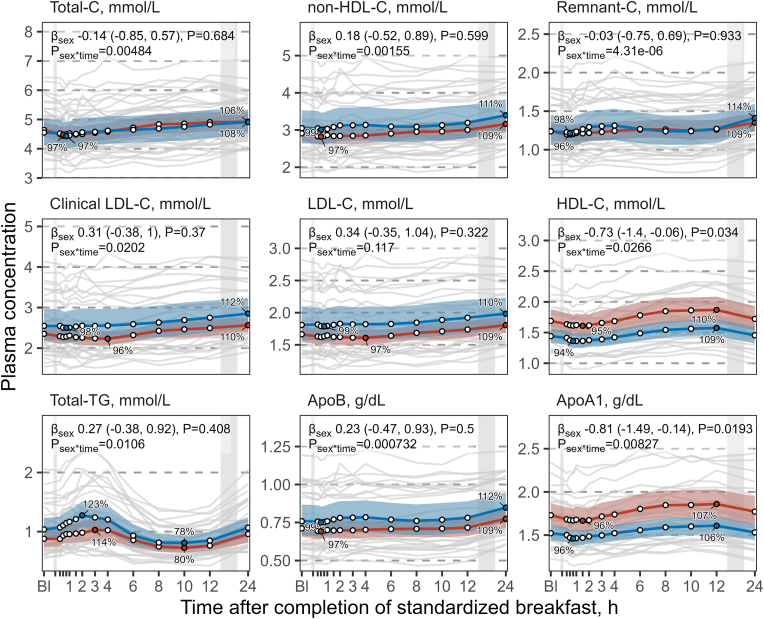
Standard lipid panel *The plasma concentrations of standard lipid biomarkers as a function of time since completion of the standardized breakfast*. The solid red and blue lines represent the geometric mean for females (n = 16) and males (n = 18), respectively, while the shaded areas represent the 95 % geometric confidence intervals (gCI). Individual levels are depicted as grey lines. The leftmost grey vertical line indicates the time of the standardized breakfast meal, while the rightmost grey vertical line indicates time spent outside the study center. Percentage of baseline are shown as average sex-specific minimum and maximum values. Sex differences (males compared to females) are shown as standard deviation (SD)-normalized β coefficients, 95 % CIs, and P values. Due to the SD-normalization, the coefficients can be compared across panels. The P values for the sex∗time interaction are derived from an ANOVA of the underlying non-linear model (see Methods for model description). Note the concentration differences for LDL-C and Clinical LDL-C; while the former is the “size-specific LDL-C″, the latter has been calibrated to routine clinical chemistry (see Methods for additional information). Abbreviations: apoB, apolipoprotein B; apoA1, apolipoprotein A1; Bl, baseline; C, cholesterol; h, hours; HDL, high density lipoprotein; LDL, low density lipoprotein; TG, triglycerides.

We previously explored the postprandial and postabsorptive changes for a standard lipid panel using clinical chemistry; here, we present standard lipid panel biomarkers analyzed by NMR metabolomics. Total-TG had the most pronounced changes during the 24-h period (+18% at 3 h, −21% at 10 h), and HDL-C and ApoA1 showed a pattern that was the inverse of Total-TG (−5% at 1.5 h, +9% at 12 h) (Fig. 1, Tables S1 and S2). Postabsorptive baseline levels were reached at 12-24 h for Total-TG, HDL-C and ApoA1. In contrast, LDL-C and LDL-C-correlated biomarkers showed minor changes in the early postprandial phase (first 6 h) but increased slightly with increased time since the meal (+9% for LDL-C at 24 h) (Fig. 1). Responses were generally similar between sexes, although males seemed to show a larger response in the early postprandial phase (Fig. 1).

### 3.3 Lipoprotein subclass particles and lipid content

**Fig. 2:**
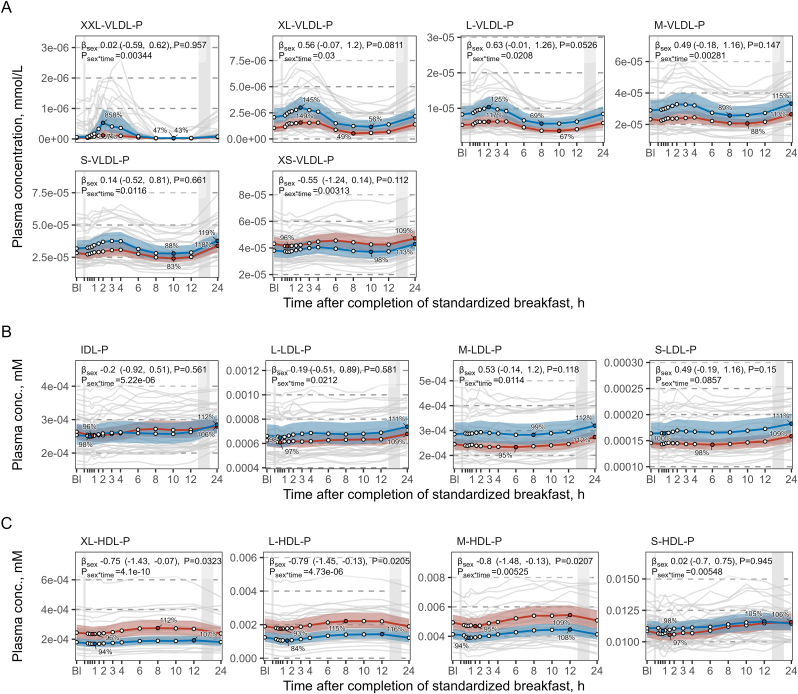
Lipoprotein subclass particles *The plasma concentrations of lipoprotein subclass particles as a function of time since completion of the standardized breakfast*. The figure shows VLDL particles (A), IDL and LDL particles (B) and HDL particles (C). See legend to Fig. 1 for a comprehensive description. Abbreviations: Bl, baseline; HDL, high density lipoprotein; h, hours; IDL, intermediate density lipoprotein; L, large; LDL, low density lipoprotein; M, medium; mM, mmol/L; P, particle; S, small; VLDL, very low-density lipoprotein; XL, extra-large; XS, extra-small; XXL, extremely large.

The lipoprotein subclasses showed different plasma levels (Fig. 2, Tables S1 and S2); for example, the gMean baseline concentrations were 0.0067 μM for L-VLDL-P, 0.64 μM for L-LDL-P (95-fold higher than L-VLDL-P), and 1.5 μM for L-HDL-P (223-fold higher than L-VLDL-P). Compared to females, males generally had higher levels of most VLDL and LDL particles, and lower levels of most HDL particles.

**Figure S1:**
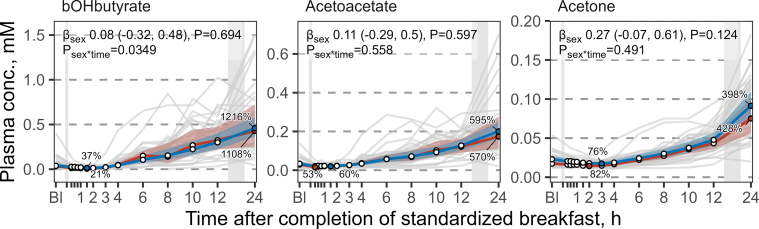
Lipid content in lipoprotein subclass particles *The plasma concentrations of main lipid types as a function of time since completion of the standardized breakfast*. The solid red and blue lines represent the geometric mean for females (n = 16) and males (n = 18), respectively, while the shaded areas represent the 95 % geometric confidence intervals (CI). Individual levels are depicted as grey lines. The leftmost grey vertical line indicates the time of the standardized breakfast meal, while the rightmost grey vertical line indicates time spent outside the study center. Percentage of baseline are shown as average sex-specific minimum and maximum values. Sex differences (males compared to females) are shown as standard deviation (SD)-normalized β coefficients, 95 % CIs, and P values. Due to the SD-normalization, the coefficients can be compared across panels. The P values for the sex∗time interaction are derived from an ANOVA of the underlying non-linear model (see Methods for model description). Abbreviations: Bl, baseline; CE, cholestetylester; FC, free cholesterol; HDL, high density lipoprotein; IDL, intermediate density lipoprotein; L, large; LDL, low density lipoprotein; M, medium; mM, mmol/L; P, particle; PL, phospholipids; S, small; TG, triglycerides; VLDL, very low-density lipoprotein; XL, extra-large; XS, extra-small; XXL, extremely large.

The VLDL subclass particles (Fig. 2A) and lipid content (Fig. S1A) showed patterns similar to Total-TG (Fig. 1). L-VLDL-P, for example, peaked at 2 h (+21%) and were lowest at 8-10 h (−32%), before increasing to baseline again towards 12-24 h (+5% at 24 h). While males had a slightly stronger VLDL increase in the early postprandial phase than females, responses were in general similar.

The IDL- and LDL subclass particles (Fig. 2B) and lipid content (Fig. S1B) were characterized by a pattern similar to Total- and LDL-C (Fig. 1); for example, L-LDL-P showed minimal change in the postprandial phase but increased slightly in the fasting state (+10% at 24 h) (Fig. 2B). Females had higher LDL-TG and lower VLDL-TG. Responses were generally similar between sexes, except for IDL-P where sex-differences were observed despite absolute levels being numerically very similar.

For the HDL subclass particles (Fig. 2C) and lipid content (Fig. S1C), we observed a trajectory similar to HDL-C and ApoA1 (Fig. 1). HDL-P, for example, decreased to the lowest concentration after 1 h (−11%), and then peaked at 8-12 h (+15%) before returning to baseline levels again at 12-24 h. Interestingly, HDL-TG and LDL-TG had a trajectory resembling Total-TG and VLDL subclass particles. Responses were again fairly similar for males and females, but HDL-TG was higher in females compared to males.

### 3.4 Inflammatory markers

**Fig. 3:**
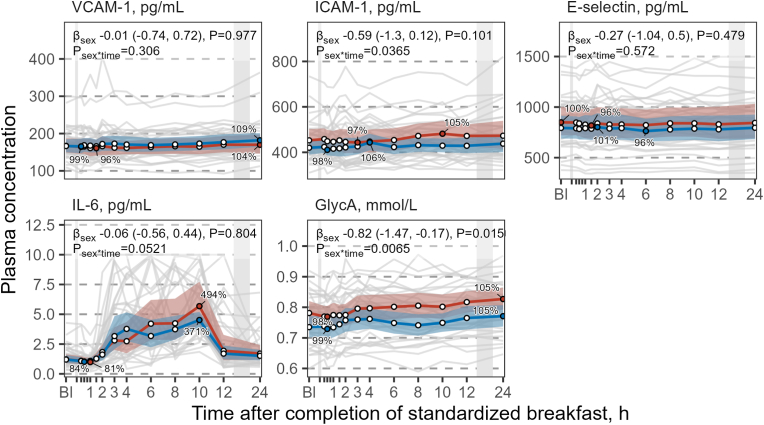
Inflammatory markers *The plasma concentrations of inflammatory markers as a function of time since completion of the standardized breakfast*. See legend to Fig. 1 for a comprehensive description. ELISA data for timepoint number 4 (45 min after baseline) was skewed due to being run on a separate plate and were therefore removed from the data set. Abbreviation: Bl, baseline.

We observed minimal changes for most of the inflammatory biomarkers (Fig. 3). gMean (95 % CI) of baseline concentrations were 170 (150, 180) pg/mL for VCAM-1, 440 (410, 460) pg/mL for ICAM-1, 820 (740, 910) pg/mL for E-selectin, 1.2 (0.99, 1.5) pg/mL for IL-6, and 0.76 (0.73, 0.78) mmol/L for GlycA. Concentrations of GlycA, VCAM-1, and ICAM-1 exhibited a slight increase at 24 h (+4-7%), while IL-6 exhibited the largest postprandial increase, with an average peak at 10 h (+325%), before returning to baseline at 12-24 h. There were no pronounced sex differences in response over time for the inflammatory biomarkers. GlycA was on average 0.8 SD lower among males compared to females.

### 3.5 Ketone bodies

**Figure S2:**
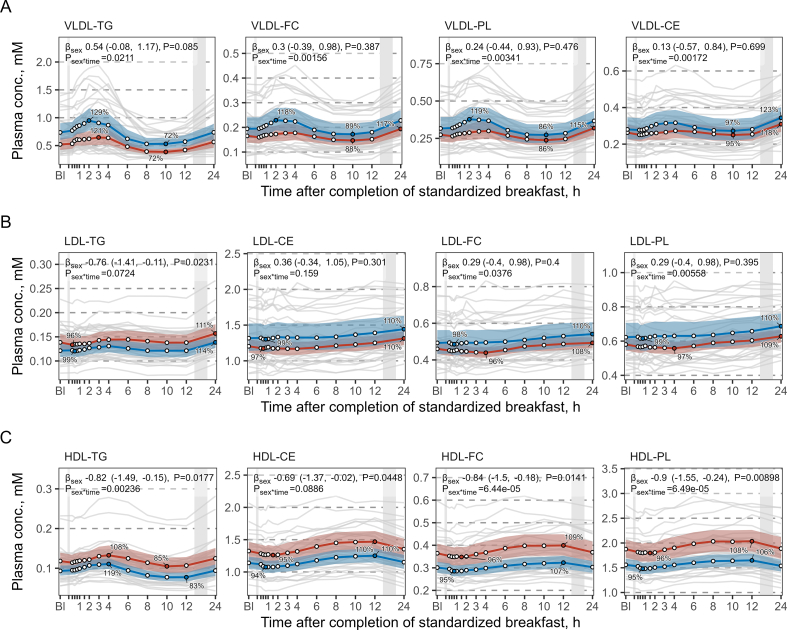
Ketone bodies *The plasma concentrations of ketone bodies as a function of time since completion of the standardized breakfast*. The solid red and blue lines represent the geometric mean for females (n = 16) and males (n = 18), respectively, while the shaded areas represent the 95 % geometric confidence intervals (CI). Individual levels are depicted as grey lines. The leftmost grey vertical line indicates the time of the standardized breakfast meal, while the rightmost grey vertical line indicates time spent outside the study center. Percentage of baseline are shown as average sex-specific minimum and maximum values. Sex differences (males compared to females) are shown as standard deviation (SD)-normalized β coefficients, 95 % CIs, and P values. Due to the SD-normalization, the coefficients can be compared across panels. The P values for the sex∗time interaction are derived from an ANOVA of the underlying non-linear model (see Methods for model description). Abbreviations: Bl, baseline; mM; millimole per liter.

Increased levels of ketone bodies confirmed that the participants were compliant with the study protocol. Ketone concentrations dropped rapidly following food intake (lowest point of −71% at 1.5h for bOHbutyrate for females and males combined, see Tables S1 and S2), and subsequently increased substantially (peak of +1167% at 24h for bOHbutyrate) (Fig. S2, Tables S1 and S2).

## 4 Discussion

In the present study of healthy young adults, we conducted a comprehensive assessment of the postprandial and fasting lipoprotein metabolism and inflammatory response over 24 h following intake of a standardized, habitual breakfast meal. Overall, food intake and fasting induced small-to-moderate changes in plasma concentrations of most biomarkers. There was prominent inter-individual variation in baseline plasma concentrations and time of return to baseline; although responses were generally similar, there was some evidence of sex differences in response for some biomarkers.

First, except for VLDL particles and TG-rich lipoproteins, most markers remained relatively stable throughout the 24-h measuring period. Therefore, when measured by NMR spectroscopy, the timing of blood sampling may be more crucial for VLDL particles and TG-rich and -related biomarkers than for the other lipid markers. Herein, participants consumed a mixed meal, and one would expect different postprandial responses depending on lipid dose ingested [21,22]. However, similar changes in lipoproteins in the postprandial and postabsorptive phases are also reported by Bermingham et al. [23] and Hansson et al. [10], despite the subjects consuming a high-carbohydrate or high-fat breakfast.

Baseline plasma concentrations varied substantially between individuals, while most tended to have similar response over time. Males and females generally had similar postprandial responses, although we observed some small differences; for example, males generally had higher concentrations for most lipoprotein particles and larger responses than females, except for HDL particles, where females tended to have higher concentrations and larger responses. These observations were also noted by Bermingham et al. [23]. It should be emphasized that we herein included healthy subjects, and that disease states [24] and genetic variation [25] typically alter baseline level and response of plasma lipids, such as TGs.

In addition to the trial evidence, several large-scale, population-based studies support that most plasma lipids and lipoproteins exhibit only modest postprandial changes and are considered clinically insignificant [4,5]. When comparing self-reported non-fasting and fasting lipid profiles, minor increases are typically found for plasma triglycerides (+0.3 mmol/L), and slight decreases in total and LDL cholesterol levels (−0.2 mmol/L), while HDL cholesterol levels are similar [4,5]. Similarly, in a cross-sectional analysis of 25,656 individuals from the general population, self-reported time since last meal was associated with subtle increases in triglyceride content and decreases in cholesterol content of lipoprotein particles [13].

We observed that several of the markers used to assess cardiovascular disease risk were highest after 24 h of fasting, such as LDL-C and ApoB. Because our sampling was limited to 24 h after food intake, we can only speculate their further trajectory had fasting been maintained for longer, as both short- and long-term fasting may impact lipid levels, depending on the context. For example, short-term Ramadan fasting in healthy subjects may reduce lipid levels [26], while clinical conditions such as anorexia nervosa may increase lipid levels [27]. Fasting-mimicking diets are also increasingly popular, and such diets may increase plasma lipid levels [28].

Taken together, our findings support that when conducting comprehensive NMR metabolomics analyses of plasma lipids and lipoproteins, time since ingestion of the meal had small to moderate influence on plasma levels over a period of 24 h.

Ingestion of food induces a physiological inflammatory response in the postprandial phase, likely driven in part by exposure to dietary lipids [[29], [30], [31]]. In our study, VCAM-1, ICAM-1, and E-selectin were unaffected in the postprandial and fasting period, which could be related to the size and the composition of the breakfast meal, or that the inflammatory response is highly specific to other pathways of the immune system. Among previous studies on the postprandial effect of high-fat meals, some have reported increased levels of ICAM-1 [32,33], while others report no change over time for either of these biomarkers [34,35]. GlycA, a biomarker that reflect the overall level and complexity of the acute phase proteins, showed an unremarkable and modest increase with fasting [36], in line with previous findings by Mazidi et al. [31]. Taken together, for VCAM-1, ICAM-1, E-selectin and GlycA, we found no strong evidence that fasting status affects their plasma level to any meaningful degree over a period of 24 h [14].

In contrast and interestingly, IL-6 showed a remarkably higher level (+325% at 10 h) after the meal compared to the fasting state at baseline. IL-6 has previously been reported to increase with age and following food intake, especially high-fat meals [30]. Also, exercise induces an increase in IL-6, especially endurance exercise [37,38]. Plasma IL-6 levels are important as they associate with future cardiovascular events in the general population both in observational studies [[39], [40], [41]] and mendelian randomization studies [42]; also, IL-6 receptor antagonism by tocilizumab increased myocardial salvage in patients with acute ST elevation myocardial infarction [43], potentially driven by inhibition of the downstream mediator CXCL10 [44]. The evidence for IL-6 being causally involved in atherosclerotic cardiovascular disease is thus accumulating.

The IL-6 concentrations observed are generally within the range reported for healthy populations. In healthy adults, mean fasting IL-6 levels are typically reported below approximately 5-7 pg/mL, and often in the range 0.5-1.8 pg/mL [31,40,41], which aligns well with the mean fasting baseline level in our study (1.2 pg/mL). At the same time, there is substantial variability across populations and studies. For instance, in a meta-analysis of 3166 healthy individuals aged 18-90 years, Said et al. reported a pooled average IL-6 concentration of 5.2 pg/mL (95% CI 4.6-5.7), with individual values ranging from 0 to 43.5 pg/mL [45]. Interestingly, in the MESA cohort, mean IL-6 levels were 0.65, 1.21, and 2.32 pg/mL across tertiles 1-3, respectively, and higher IL-6 levels were associated with increased risk of all-cause, cardiovascular, and non-cardiovascular mortality, as well as incident heart failure [46].

Taken together, the inflammatory response to diet is variable across biomarkers, and time since last meal may be of importance for certain inflammatory biomarkers, such as IL-6, although this must be further studied.

Given that the study participants were healthy, young subjects, the observed changes in lipoprotein metabolism and inflammation can likely be explained as normal physiology. The postprandial response likely reflects absorption and redistribution of lipids from enterocytes to the liver and peripheral tissues, whereas the fasting response is likely driven by gradual depletion of hepatic glycogen and ensuing metabolic adjustments to preserve plasma glucose, including increased adipose tissue lipolysis which delivers free fatty acids to the liver for ketogenesis or re-esterification to triglycerides and subsequent redistribution to peripheral tissues [1]. Interestingly, IL-6 may also play a role in the ongoing energy redistribution by directly regulating lipid metabolism in both fed and fasted states, for example by promoting lipolysis [38,47]. Circadian rhythms also influence lipid handling and inflammation and could partly account for changes across postprandial and fasting phases, but our study design does not allow assessment of their relative contribution [48,49].

Finally, biomarker concentrations returned to postabsorptive baseline levels at different time points for different biomarkers. Again, interindividual differences were noted, but on average, baseline levels were reached at 12-24 h for VLDL and HDL particles and IL-6. In contrast, IDL and LDL particles and other inflammatory biomarkers may be higher than baseline at 24 h.

While the main aim of the present work was to characterize physiological changes, our results may also have practical implications. First, our data may serve as reference material for converting between fasting and non-fasting values of lipoproteins and inflammatory biomarkers in healthy young individuals. Second, the high temporal resolution of our measurements could inform more cost-effective data collection strategies in future fasting and postprandial studies. Finally, non-fasting lipid levels are now increasingly used in clinical decision making [5]. Current guidelines are based on large population studies with self-reported time since last meal; here, we provide evidence that supports these population-based findings at much higher temporal resolution and with detailed individual-level trajectories over 24 h.

### 4.1 Strengths and limitations

The main strengths of the current study are the repeated sampling within individuals over a long duration of 24 h, including both males and females, collected in a well-controlled environment. The relatively homogenous study population, and standardized procedures, limits the external sources of variation such as due to age, health status, body composition, or physical activity. Of note, the standardized evening meal before the overnight fast limited the expected variation in the second-meal effect [50]. Further, the high frequency of samples provides accurate data on the dynamic changes in metabolite concentrations during the study period, and the sample size was large enough to achieve sufficient precision. A comprehensive panel of lipid and inflammatory biomarkers allowed for a detailed description of the dynamics of the postprandial and postabsorptive lipid metabolism and inflammation. The compliance was good, as evidenced by the expected patterns in ketone, insulin, and glucose concentrations [15]. Furthermore, strict time control limited any effect of day variation.

Some limitations warrant attention. Despite measures taken to limit variations due to other factors and maximize the internal validity, there were still some variations regarding body composition and estimated resting metabolic rate. These may have affected the postprandial responses and introduced some variability. Further, the standardized design and the artificial setting limits the external validity, and our ability to generalize to other contexts such as other age groups, non-healthy individuals, other meal composition or portion sizes, different prior food intake [50], and meals at other times of the day. For example, diabetes may lead to disturbed lipid metabolism characterized by delayed concentration peak and prolonged high concentration of plasma triglycerides and VLDL particles [51]. Also, while responses were significantly different between sexes for many biomarkers (P_sex∗time_ in Figures and Table S3), responses were generally similar for age and BMI groups, except for a subset of biomarkers, mainly ICAM-1 (P_BMI∗time_<0.001), VCAM-1 (P_BMI∗time_<0.01), and L-LDL-P (P_age∗time_<0.01) (Table S3). However, interaction P values for age and BMI should be interpreted with caution due to the restricted distribution of these covariates in our sample. Our trajectories should thus primarily be viewed as high-resolution reference patterns for young, healthy adults, and more research is needed among other life-stage groups and more metabolically diverse populations.

Taken together, our results are not directly applicable to the general healthy population across life-stage groups; while prior evidence demonstrate fairly similar relative changes over time in both men [1] and women [52], limitations in previous literature include small sample sizes, analyte specific-differences, and an overall lack of granular data on age-related differences.

Finally, although we have many measurements, especially in the early postprandial phase, we lack measurement points between 12 and 24 h, which limits our resolution in this part of the fasting period. Also, hormonal changes in females due to menstrual cycle were not considered.

## 5 Conclusions

In conclusion, there was inter-individual variation in baseline plasma concentrations and time of return to baseline. Although responses were generally similar, we found evidence of sex differences in response for some biomarkers. While TG-rich lipoproteins and VLDL particles increased in the postprandial phase and returned to baseline at 12-24 h, IDL and LDL particles showed small changes in the postprandial and early postabsorptive phases and then increased at 24 h. Similarly, while most inflammatory markers showed small changes, we observed a substantial increase in IL-6 during the first 10 h after food intake, which returned to baseline at 12-24 h.

## CRediT author statement

Silje-Marie Jensen: Conceptualization, Formal analysis, Investigation, Methodology, Validation, Visualization, Writing – original draft, Writing – review & editing.

Kirsten B. Holven: Conceptualization, Resources, Supervision, Validation, Writing – review & editing.

Stine M. Ulven: Conceptualization, Resources, Supervision, Validation, Writing – review & editing.

Åslaug Matre Anfinsen: Data curation, Investigation, Methodology, Project administration, Validation, Writing – review & editing.

Jutta Dierkes: Funding acquisition, Investigation, Methodology, Resources, Supervision, Validation, Writing – review & editing.

Vegard Lysne: Data curation, Funding acquisition, Investigation, Methodology, Project administration, Resources, Supervision, Validation, Writing – review & editing.

Jacob J. Christensen: Data curation, Formal analysis, Investigation, Methodology, Project administration, Resources, Software, Supervision, Validation, Visualization, Writing – original draft, Writing – review & editing.

## Declaration of generative AI and AI-assisted technologies in the writing process

During the preparation of this work the authors used GPT UiO in order to improve readability and language. After using this tool, the authors reviewed and edited the content as needed and take full responsibility for the content of the publication.

## Sources of support

The Postprandial Metabolism study's data collection was performed at the Research Unit for Health Surveys at the University of Bergen, which received funding from the Trond Mohn Foundation (grant ID BFS2017TMT02). The study was part of the Mohn Nutrition Research Laboratory, which received support from the Trond Mohn Foundation, the University of Bergen and Haukeland University Hospital (2018-2023). The present analysis was conducted at the University of Oslo, with additional support from the Throne-Holst Foundation for Nutrition Research and the University of Oslo.

## Conflicts of interest

All authors have completed the ICMJE uniform disclosure form and declare the following conflicts of interest. Dr. Holven has received personal fees from Sanofi and Ultragenyx, none of which are related to the content of this manuscript. Dr. Christensen has received personal fees from Novo Nordisk and Falck Helse, not related to the content of this manuscript. The other authors have no financial relationships relevant to disclose.
